# Fully automatic estimation of global left ventricular systolic function using deep learning in transoesophageal echocardiography

**DOI:** 10.1093/ehjimp/qyad007

**Published:** 2023-07-04

**Authors:** Erik Andreas Rye Berg, Anders Austlid Taskén, Trym Nordal, Bjørnar Grenne, Torvald Espeland, Idar Kirkeby-Garstad, Håvard Dalen, Espen Holte, Stian Stølen, Svend Aakhus, Gabriel Kiss

**Affiliations:** Centre for Innovative Ultrasound Solutions, Department of Circulation and Medical Imaging, Faculty of Medicine and Health Science, Norwegian University of Science and Technology, Prinsesse Kristinas gate 3, Trondheim 7030, Norway; Department of Circulation and Medical Imaging, Faculty of Medicine and Health Science, Norwegian University of Science and Technology, Prinsesse Kristinas gate 3, Trondheim 7030, Norway; Clinic of Cardiology, St Olavs Hospital, Trondheim University Hospital, Prinsesse Kristinas gate 3, Trondheim 7030, Norway; Department of Computer Science, Faculty of Information Technology and Electrical Engineering, Norwegian University of Science and Technology, Trondheim 7034, Norway; Department of Engineering Cybernetics, Faculty of Information Technology and Electrical Engineering, Norwegian University of Science and Technology, Trondheim 7034, Norway; Department of Circulation and Medical Imaging, Faculty of Medicine and Health Science, Norwegian University of Science and Technology, Prinsesse Kristinas gate 3, Trondheim 7030, Norway; Clinic of Cardiology, St Olavs Hospital, Trondheim University Hospital, Prinsesse Kristinas gate 3, Trondheim 7030, Norway; Department of Circulation and Medical Imaging, Faculty of Medicine and Health Science, Norwegian University of Science and Technology, Prinsesse Kristinas gate 3, Trondheim 7030, Norway; Clinic of Cardiology, St Olavs Hospital, Trondheim University Hospital, Prinsesse Kristinas gate 3, Trondheim 7030, Norway; Department of Circulation and Medical Imaging, Faculty of Medicine and Health Science, Norwegian University of Science and Technology, Prinsesse Kristinas gate 3, Trondheim 7030, Norway; Department of Anaesthesiology and Intensive Care Medicine, St Olavs Hospital, Trondheim University Hospital, Trondheim 7030, Norway; Department of Circulation and Medical Imaging, Faculty of Medicine and Health Science, Norwegian University of Science and Technology, Prinsesse Kristinas gate 3, Trondheim 7030, Norway; Clinic of Cardiology, St Olavs Hospital, Trondheim University Hospital, Prinsesse Kristinas gate 3, Trondheim 7030, Norway; Clinic of Cardiology, St Olavs Hospital, Trondheim University Hospital, Prinsesse Kristinas gate 3, Trondheim 7030, Norway; Clinic of Cardiology, St Olavs Hospital, Trondheim University Hospital, Prinsesse Kristinas gate 3, Trondheim 7030, Norway; Department of Circulation and Medical Imaging, Faculty of Medicine and Health Science, Norwegian University of Science and Technology, Prinsesse Kristinas gate 3, Trondheim 7030, Norway; Clinic of Cardiology, St Olavs Hospital, Trondheim University Hospital, Prinsesse Kristinas gate 3, Trondheim 7030, Norway; Department of Computer Science, Faculty of Information Technology and Electrical Engineering, Norwegian University of Science and Technology, Trondheim 7034, Norway

**Keywords:** deep learning, artificial intelligence, transoesophageal echocardiography, systolic function, mitral annular plane systolic excursion, monitoring

## Abstract

**Aims:**

To improve monitoring of cardiac function during major surgery and intensive care, we have developed a method for fully automatic estimation of mitral annular plane systolic excursion (auto-MAPSE) using deep learning in transoesophageal echocardiography (TOE). The aim of this study was a clinical validation of auto-MAPSE in patients with heart disease.

**Methods and results:**

TOE recordings were collected from 185 consecutive patients without selection on image quality. Deep-learning-based auto-MAPSE was trained and optimized from 105 patient recordings. We assessed auto-MAPSE feasibility, and agreement and inter-rater reliability with manual reference in 80 patients with and without electrocardiogram (ECG) tracings. Mean processing time for auto-MAPSE was 0.3 s per cardiac cycle/view. Overall feasibility was >90% for manual MAPSE and ECG-enabled auto-MAPSE and 82% for ECG-disabled auto-MAPSE. Feasibility in at least two walls was ≥95% for all methods. Compared with manual reference, bias [95% limits of agreement (LoA)] was −0.5 [−4.0, 3.1] mm for ECG-enabled auto-MAPSE and −0.2 [−4.2, 3.6] mm for ECG-disabled auto-MAPSE. Intra-class correlation coefficient (ICC) for consistency was 0.90 and 0.88, respectively. Manual inter-observer bias [95% LoA] was −0.9 [−4.7, 3.0] mm, and ICC was 0.86.

**Conclusion:**

Auto-MAPSE was fast and highly feasible. Inter-rater reliability between auto-MAPSE and manual reference was good. Agreement between auto-MAPSE and manual reference did not differ from manual inter-observer agreement. As the principal advantages of deep-learning-based assessment are speed and reproducibility, auto-MAPSE has the potential to improve real-time monitoring of left ventricular function. This should be investigated in relevant clinical settings.

## Graphical Abstract

**Graphical Abstract:**
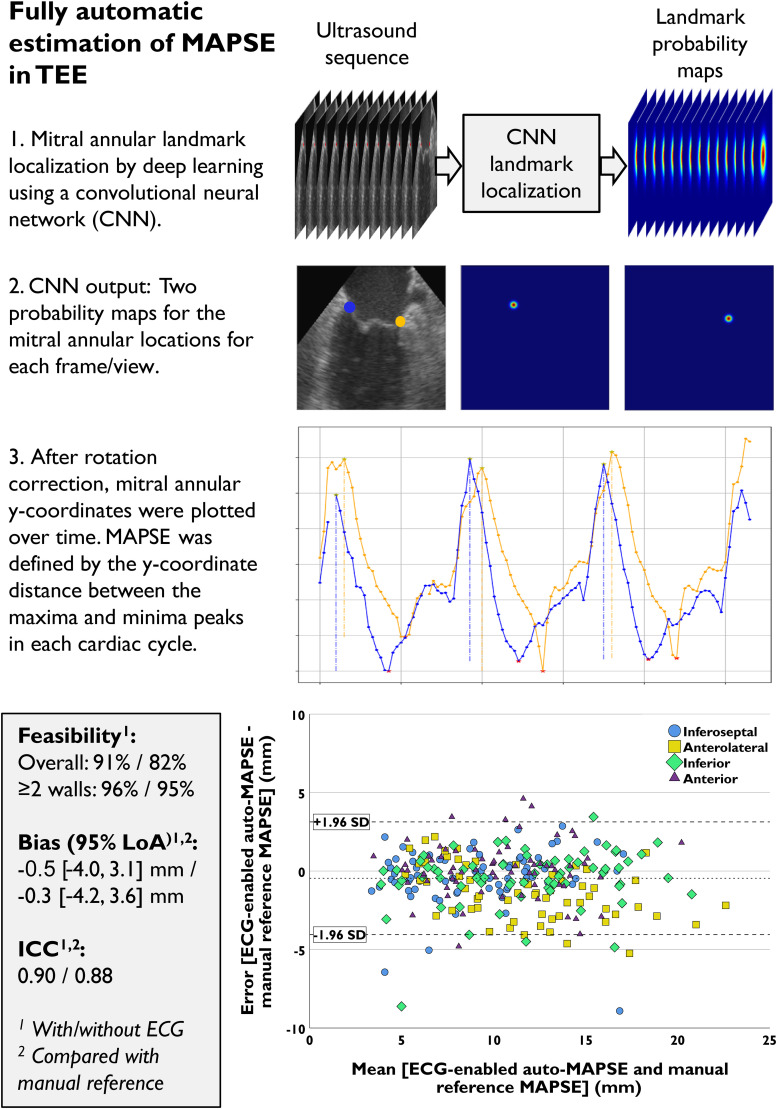
Auto-MAPSE, automatic estimation of mitral annular plane systolic excursion; CNN, convolutional neural network; ECG, electrocardiogram; ICC, intra-class correlation coefficient; LoA, limits of agreement; MAPSE, mitral annular plane systolic excursion; SD, standard deviation; TOE, transoesophageal echocardiography.

## Introduction

Cardiac complications in relation to major surgery and intensive care are common.^1–4^ Prompt detection and characterization of cardiac dysfunction is essential for optimal handling.

Echocardiography is of great value in intensive care.^5^ This includes first-line echocardiography to identify mechanisms of shock.^3^ Moreover, monitoring with transoesophageal echocardiography (TOE) is recommended in high-risk perioperative settings.^6,7^ Simplified intermittent TOE has been studied with promising results.^8,9^ However, true monitoring by TOE requires either continuous manual interaction or fully automatic methods. To date, such methods have not been readily available for TOE.

Deep learning, an advancement of artificial intelligence that deploys artificial neural networks, has held great promise for the automatic assessment of left ventricular (LV) systolic function in transthoracic echocardiography. Generally, the fundamental step for these methods has been LV segmentation.^10,11^ However, in TOE-based monitoring, the probe should be kept unlocked to minimize patient hazard.

Without active handling, this typically leads to foreshortened views of the cardiac chambers. The presence of air between the probe and the oesophageal wall, LV shadowing from mitral annular calcifications, and depth attenuation may be additional challenges. All these factors impair LV segmentation. Therefore, adaption of previously developed segmentation-based deep learning methods may not function optimally in TOE-based monitoring.

Importantly, mitral annular plane systolic excursion (MAPSE) does not rely on LV segmentation. Moreover, it contributes to 60–75% of the LV stroke volume.^12,13^ MAPSE is strongly correlated to global longitudinal strain^14^ and has a high feasibility, even in suboptimal image quality.^15,16^ MAPSE from TOE reliably detected LV dysfunction during off-pump cardiac surgery and intensive care.^17,18^ To improve monitoring of cardiac function during major surgery and intensive care, we have developed a method for fully automatic estimation of MAPSE (auto-MAPSE) by TOE using deep learning. The method has been developed to run both with and without electrocardiogram (ECG) tracings, as diathermy distorts the ECG.

Accordingly, the aim of this study was a clinical validation of auto-MAPSE in patients with heart disease. This included assessments of feasibility, reliability and agreement with manual reference, and the importance of ECG tracings.

## Methods

### Study population

We included TOE recordings from 185 patients investigated as part of standard clinical care at St Olavs Hospital (Trondheim, Norway), a secondary university hospital with services in interventional cardiology, electrophysiology, and cardiothoracic surgery. Standard clinical contraindications to TOE applied. There was no selection based on image quality. Patient tolerance to withstand a full TOE examination was the only selection criterium for inclusion.

A total of 180 examinations were performed consecutively by 4 experts at an echocardiography laboratory accredited by the European Association of Cardiovascular Imaging. Five examinations were recorded during open heart surgery by an experienced thoracic anaesthesiologist. The recordings were split into training, pilot validation, and clinical validation data sets (*Table 1*). Clinical validation was performed on the final 80 consecutive examinations, all recorded at the echocardiography laboratory. From these, inter-observer assessment was performed on a randomly drawn subset of 30 examinations. The sample size was defined based on our research group’s experience with the development of deep learning methods for similar applications. In this manuscript, the term validation is used synonymously to testing, of which the latter is more typically used in technical deep learning literature.

**Table 1:** Data overview

Data set	Patients, *n*	Frames total, *n*	Frames 4-chamber view, %	Frames 2-chamber view, %
Training	66	8520	50.2	49.8
Validation during training	16	1782	50.1	49.9
Pilot validation and filter customization	23	2879	49.8	50.2
Clinical validation^a^	80	17 317	50.0	50.0
Total	185	30 498	50.0	50.0

a A random sample of 30 patients was used for inter-observer assessment.

### Data acquisition

*Table 2* displays recording equipment and settings. To mimic monitoring settings, the examiners were encouraged to obtain the relevant recordings with the probe unlocked without forced flex or tilt. The patients were positioned according to the clinical setting and the preference of the examiner. No specific instructions on respiration were given. Only anonymized ultrasound data and corresponding ECG tracings were collected.

**Table 2:** Recording settings

System	GE Vivid E95/E9/S70^a^
Transducer	GE 6VT-D^a^
Software	204.xx
Application	Cardiac_E
Sector width	90 degrees
Image depth	Include full view of left ventricle
Frame rate	Default
Tilt/flex	No interaction/neutral
Cycles	3
Views	Mid-oesophageal 2- and 4-chamber views
Modes	B-mode, tissue Doppler imaging

a GE Vingmed Ultrasound, Horten, Norway.

### Automatic MAPSE estimation

**Figure 1:**
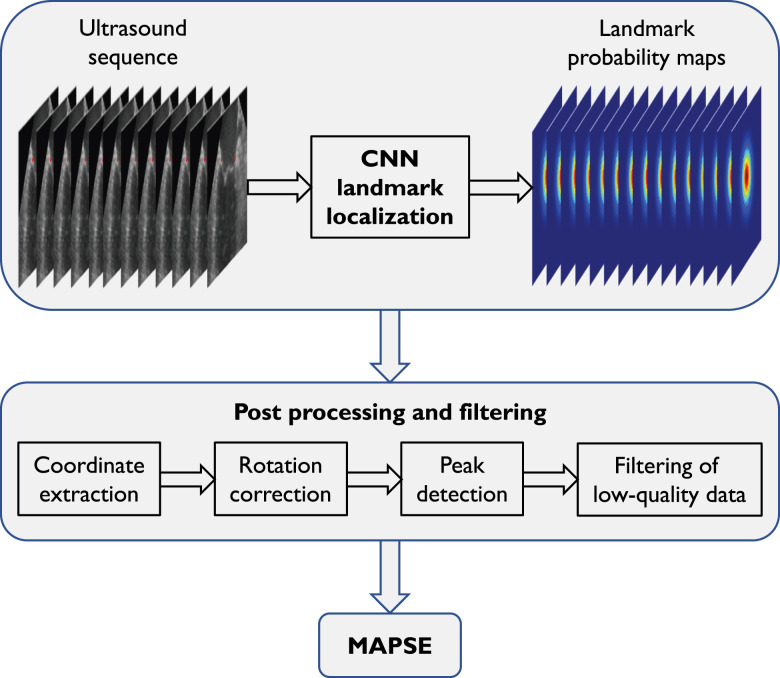
Overview of the auto-MAPSE pipeline. CNN-based landmark localization provides probability maps for the location of the mitral annulus in each frame. Following rotation correction, the landmark *y*-coordinates are plotted over time. End-diastole and end-systole are defined as the maxima and minima peaks of these plots. MAPSE is defined as the *y*-coordinate distance between these peaks. Filters are applied to discard MAPSE estimates from low-quality plots.

The pipeline for auto-MAPSE consists of deep-learning-based landmark localization, and algorithms for post processing and filtering (*Figure 1*). We ran auto-MAPSE on portable commercial hardware (11th Gen Intel® Core™ i7-11850H @ 2.50 GHz CPU and NVIDIA RTX A3000 Mobile GPU).

#### Mitral annular localization

Localization of the mitral annulus from mid-oesophageal two- and four-chamber view recordings was performed by a convolutional neural network (CNN) trained in a supervised manner. Localization was based on the identification of the mitral annular landmarks and their coordinate locations. We manually annotated the mitral annulus frame-by-frame in all 10 302 frames used for CNN training. Aiming for the mitral valve hinge points, this work was performed by two master’s students under expert guidance. Invisible mitral annular landmarks were marked ‘invisible due to noise’. To limit annotation workload, CNN training was performed on low-frame-rate B-mode extractions from tissue Doppler imaging, averaging 19 frames per second, without any visible difference in image quality from standard B-mode recordings.

A distinction between imaging views was not made during training. The CNN simply learned the mitral annular landmarks as ‘right’ and ‘left’ independent of the view, without any view detection. This was employed to reduce complexity and increase the ability to generalize. By adding random rotation and cropping, the data were augmented to effectively increase the data set size and prevent overfitting. This also prevented the CNN from learning landmark positions relative to the image sector.

**Figure 2:**
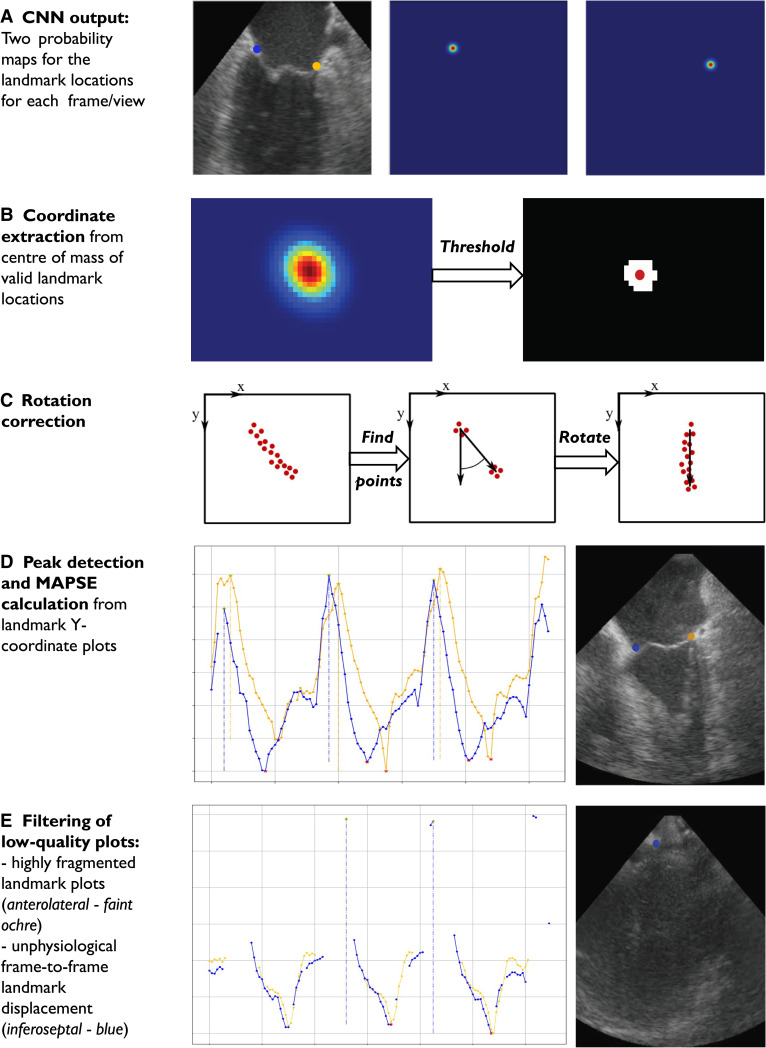
(*A–E*) Auto-MAPSE: CNN output and post-processing chain. Scales of *y*-coordinate plots in (*D*) and (*E*) are not comparable. In (E), note the poor image quality related to atrial contraction and large estimated frame-to-frame jumps due to CNN misinterpretation of proximal clutter in a few frames. In this example (*E*), all estimates from both walls were discarded by the filter algorithms.

For each ultrasound frame, the CNN generated two maps of mitral annular landmark localization, one for each wall (*Figure 2A*). Each map included probability estimates (0–100%) for the landmark localization.

The CNN was designed with a ResNet50 architecture,^19^ which was slightly modified for mitral annular localization. For further details on CNN design and settings, we refer to Nordal.^20^

#### Post-precessing and filtering

The post-processing algorithm transformed frame-by-frame CNN landmark localization to MAPSE estimates (*Figure 2*).

Mitral annular coordinates were extracted from the landmark probability maps (*Figure 2A*).

Landmark localization with a CNN-estimated probability of at least 50% was considered valid. Correspondingly, <50% probability for the landmark localization led to the discard of the specific landmark in the specific frame. The fixed 50% probability threshold, independent of acquisition settings or image quality, was determined empirically from evaluation during CNN training and assessment of the pilot validation data set (*Table 1*). Moreover, by this definition, the CNN could return more than one valid landmark location for each frame/wall. The centre of mass of all valid locations provided the final landmark location coordinates (*Figure 2B*). After rotation correction aligning mitral annular displacement to the *y*-axis, the *y*-coordinates of the estimated landmark locations were plotted over time (*Figure 2C* and *D*).

MAPSE was given by the *y*-coordinate distance between the maxima and minima peaks of the landmark *y*-coordinate plot, corresponding to end-diastole and end-systole, respectively (*Figure 2D*).

This was performed both with and without corresponding ECG tracings. For ECG-disabled auto-MAPSE, the cardiac cycle was defined by the peaks of the plot. MAPSE was estimated for each cardiac cycle and averaged over the number of valid cycles.

Customized from our investigation of the pilot validation data set (*Table 1*), we applied filters to exclude highly fragmentary landmark location plots and unphysiological frame-to-frame jumps (*Figure 2E*):

- For each wall, data from a cardiac cycle were discarded if the CNN provided valid landmark localization in <60% of the frames of this cycle.
- For ECG-enabled auto-MAPSE, this was weighted around the R-wave by applying a normalized exponential distribution (weight(*x*) = *λ*exp, *λ* = 0.1).
- If the estimated frame-to-frame mitral annular displacement exceeded 5 mm, the corresponding cardiac cycle (ECG-enabled auto-MAPSE) or peak (ECG-disabled auto-MAPSE) was discarded for this wall.

Feasibility of auto-MAPSE was determined automatically from several steps of the pipeline: CNN landmark localization probability >50% in at least 60% of the frames in a cardiac cycle, automatically detectable peaks of the mitral annular plots, and absence of unphysiological auto-MAPSE estimates. Following these algorithms, non-feasibility was defined for each wall as failure to obtain at least one auto-MAPSE estimate per three-cycle recording.

### Manual reference

**Figure 3:**
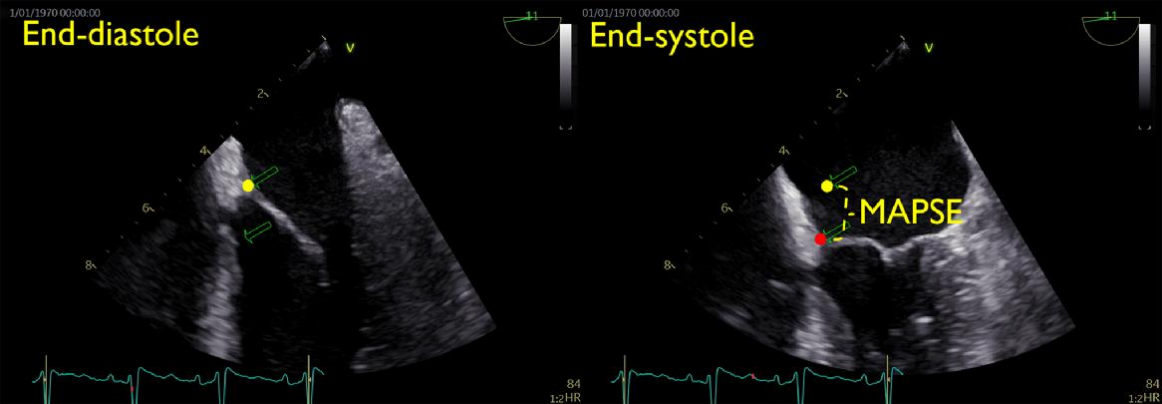
Manual MAPSE estimation. Zoomed mid-oesophageal four-chamber view at end-diastole and end-systole. MAPSE was defined as the displacement of the mitral annulus from end-diastole to end-systole for each wall.

Manual MAPSE reference was estimated using standard clinical software (EchoPac 204; GE Vingmed Ultrasound, Horten, Norway). MAPSE was defined as the maximal displacement of the mitral annulus in each cardiac cycle, measured directly on the 2D images (*Figure 3*), and averaged over the number of cycles. Invisible or indistinguishable mitral annular echo at end-diastole or end-systole was defined as non-feasibility for the specific cardiac cycle/wall. Observer 1 (E.A.R.B.) analysed the full data set and is referred to as the manual reference. Observer 2 (T.E.) analysed the inter-observer subset exclusively.

### Statistical analyses

We assessed feasibility, agreement, and inter-rater reliability for auto-MAPSE and manual observers. Residual plots and Bland–Altman plots were assessed for heteroscedasticity. Normality was investigated using histograms, QQ plots, and the Shapiro–Wilk test. Minor deviation from normality due to outliers was accepted for parametric analysis. All outliers were included in the analyses.

Descriptive measures are reported in numbers (%) or mean (±standard deviation (SD)) unless otherwise specified. We report the bias and 95% limits of agreement (LoA) of MAPSE estimates. Inter-rater reliability was assessed by a mixed-model intra-class correlation of consistency. The intra-class correlation coefficient (ICC) is reported with a 95% confidence interval (CI). For linear correlation, we report Pearson’s correlation coefficient, *r* (95% CI). Fisher’s *z*-transformation was applied to compare correlation coefficients. Differences in feasibility were assessed by Fisher’s exact test. For inter-rater reliability, linear correlation, and feasibility, *P*-values <0.05 were considered to represent statistically significant differences. Data were managed in IBM SPSS Statistics, v. 28 (IBM, New York, NY, USA).

## Results

### Data characteristics

**Figure 4:**
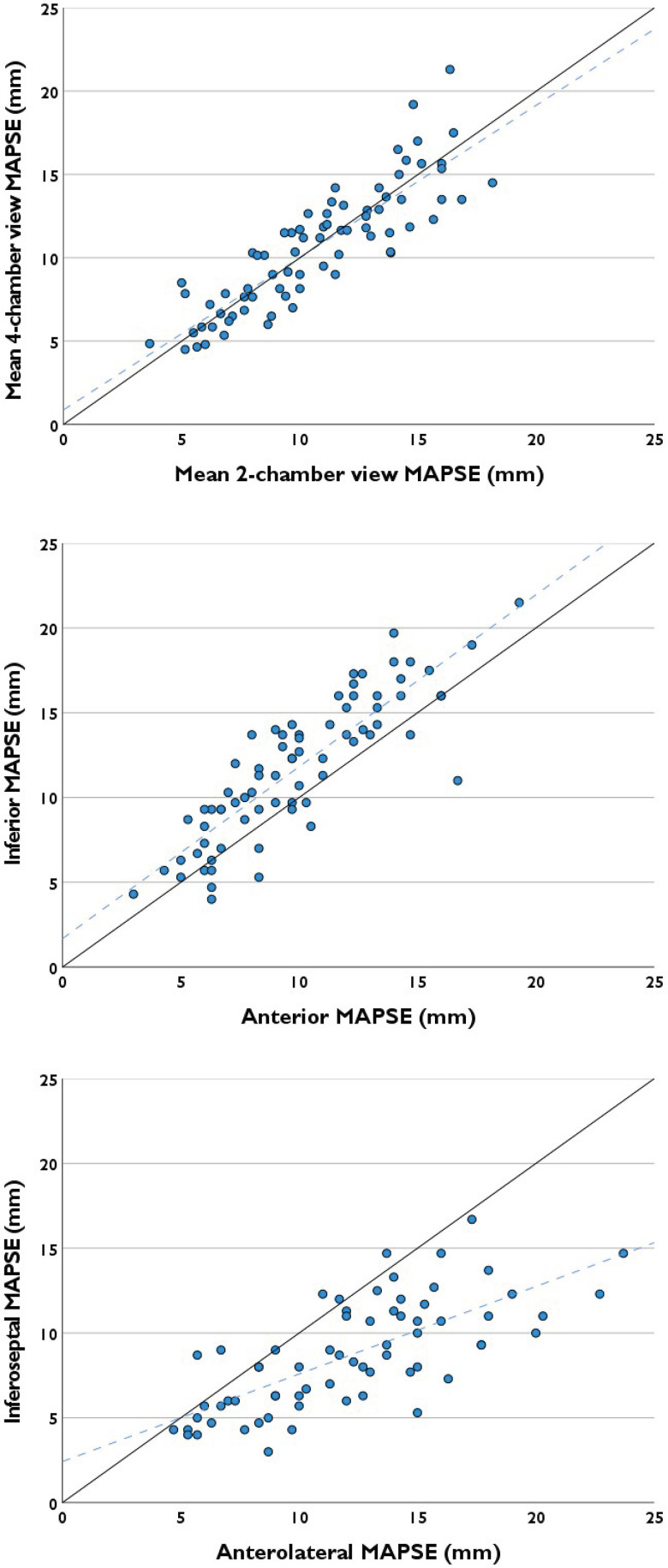
Correlation between views and walls for manual reference MAPSE. Equality and regression (dashed) lines are displayed. Mean MAPSEs from mid-oesophageal four- and two-chamber views had a strong linear correlation, *r* = 0.87 (0.80–0.91). Inferior and anterior MAPSEs had a similarly strong linear correlation, *r* = 0.86 (0.78–0.91). Compared with these, the linear correlation between inferoseptal and anterolateral MAPSEs was more moderate, *r* = 0.72 (0.58–0.82) (*P* = 0.013 and *P* = 0.025, respectively).

For the clinical validation, we included mid-oesophageal 4- and 2-chamber view recordings over 3 cardiac cycles from 80 patients. Data characteristics are displayed in *Table 3*. Arrhythmia was present in 17 (21%) of the recordings. Heart rate ranged from 42 to 153 beats per minute. Single-wall MAPSE by manual reference ranged from 3.0 to 23.7 mm. *Figure 4* displays correlation of manual reference MAPSE between views and walls. There was a strong correlation between mean four- and two-chamber view MAPSEs and between inferior and anterior MAPSEs. Processing time for auto-MAPSE averaged 0.3 s per cardiac cycle/view.

**Table 3:** Data characteristics^a^

Heart rate,/min		74 (±20)
	Minimum—maximum	42–153
Arrhythmia during recordings	Atrial fibrillation	12 (15%)
	Atrial flutter	4 (5%)
	Premature ventricular complex	1 (1%)
Frame rate,/s		38 (±6)
Manual MAPSE, single wall, mm		10.7 (±4.0)
	Minimum—maximum	3.0–23.7
Manual MAPSE processing time, s	Per 3-cycle recording	139 (±33)
Auto-MAPSE processing time, s	Per frame	0.009 (±0.0009)
	Per 3-cycle recording	0.99 (±0.26)

a Values are reported as mean (±SD) or *n* (%) unless otherwise stated.

### Feasibility

Feasibility of MAPSE estimation is presented in *Table 4*. Overall feasibility was 92% for manual reference and 91% for ECG-enabled auto-MAPSE. Overall feasibility for ECG-disabled auto-MAPSE was 82%, which was significantly lower than for manual reference and ECG-enabled auto-MAPSE.

**Table 4:** Feasibility, agreement, and inter-rater reliability in full data set (*n* = 80)

Analysis	Wall	ECG-enabled auto-MAPSE	ECG-disabled auto-MAPSE	Manual reference
Feasibility, *n* (%)	Overall (4 walls)	290 (91%)^a^	263 (82%)^a,b^	294 (92%)^a,b^
	Inferoseptal	73 (91%)	64 (80%)^c^	77 (96%)^c,d^
	Anterolateral	71 (89%)	63 (79%)	67 (84%)^d,e^
	Inferior	74 (93%)	66 (83%)	74 (93%)
	Anterior	72 (90%)	70 (88%)	76 (95%)^e^
	≥1 wall feasible	77 (96%)	77 (96%)	78 (98%)
	≥2 walls feasible	77 (96%)	76 (95%)	76 (95%)
	≥1 view (both walls feasible)	72 (90%)	68 (85%)	74 (93%)
	All 4 walls feasible	64 (80%)^f^	45 (56%)^f,g^	67 (84%)^g^
Feasibility provided feasible reference, *n* (%)	Overall (4 walls)	276 (94%)^h^	256 (87%)^h^	
	Inferoseptal	71 (92%)	63 (82%)	
	Anterolateral	64 (96%)	58 (87%)	
	Inferior	71 (96%)	66 (89%)	
	Anterior	70 (92%)	69 (91%)	
Bias [95% limits of agreement], mm*	Overall (4 walls)	−0.5 [−4.0, 3.1]	−0.3 [−4.2, 3.6]	
	Inferoseptal	−0.3 [−3.9, 3.3]	−0.1 [−3.5, 3.4]	
	Anterolateral	−1.3 [−4.7, 2.1]	−1.2 [−6.1, 3.6]	
	Inferior	−0.4 [−3.9, 3.1]	−0.1 [−2.8, 2.6]	
	Anterior	0.1 [−3.3, 3.5]	0.3 [−3.6, 4.2]	
Intra-class correlation coefficient for consistency (95% confidence interval)*	Overall (4 walls)	0.90 (0.87–0.92)	0.88 (0.85–0.90)	
	Inferoseptal	0.88 (0.81–0.92)	0.88 (0.82–0.93)^i^	
	Anterolateral	0.92 (0.86–0.95)	0.82 (0.72–0.89)^j^	
	Inferior	0.92 (0.87–0.95)	0.94 (0.91–0.96)^i,j,k^	
	Anterior	0.87 (0.81–0.92)	0.85 (0.77–0.90)^k^	

Superscript letters denote pairwise statistically significant differences in feasibility or intra-class correlation between methods (same row) or between individual walls within each method (same column). ^a^ *P* = 0.003; ^b^ *P* < 0.001; ^c^*P* = 0.003; ^d^*P* = 0.015; ^e^*P* = 0.038; ^f^*P* = 0.002; ^g^*P* < 0.001; ^h^*P* = 0.007; ^i^*P* = 0.045; ^j^*P* = 0.002; ^k^*P* = 0.006 (*P* ≥ 0.05 for all other comparisons).

* Compared with manual reference.

The feasibility of at least one wall was 96% for both ECG-enabled and ECG-disabled auto-MAPSE. Feasibility in at least two walls was 96 and 95%, respectively. There were no statistically significant differences in auto-MAPSE feasibility between the individual walls.

### Agreement and inter-rater reliability

**Figure 5:**
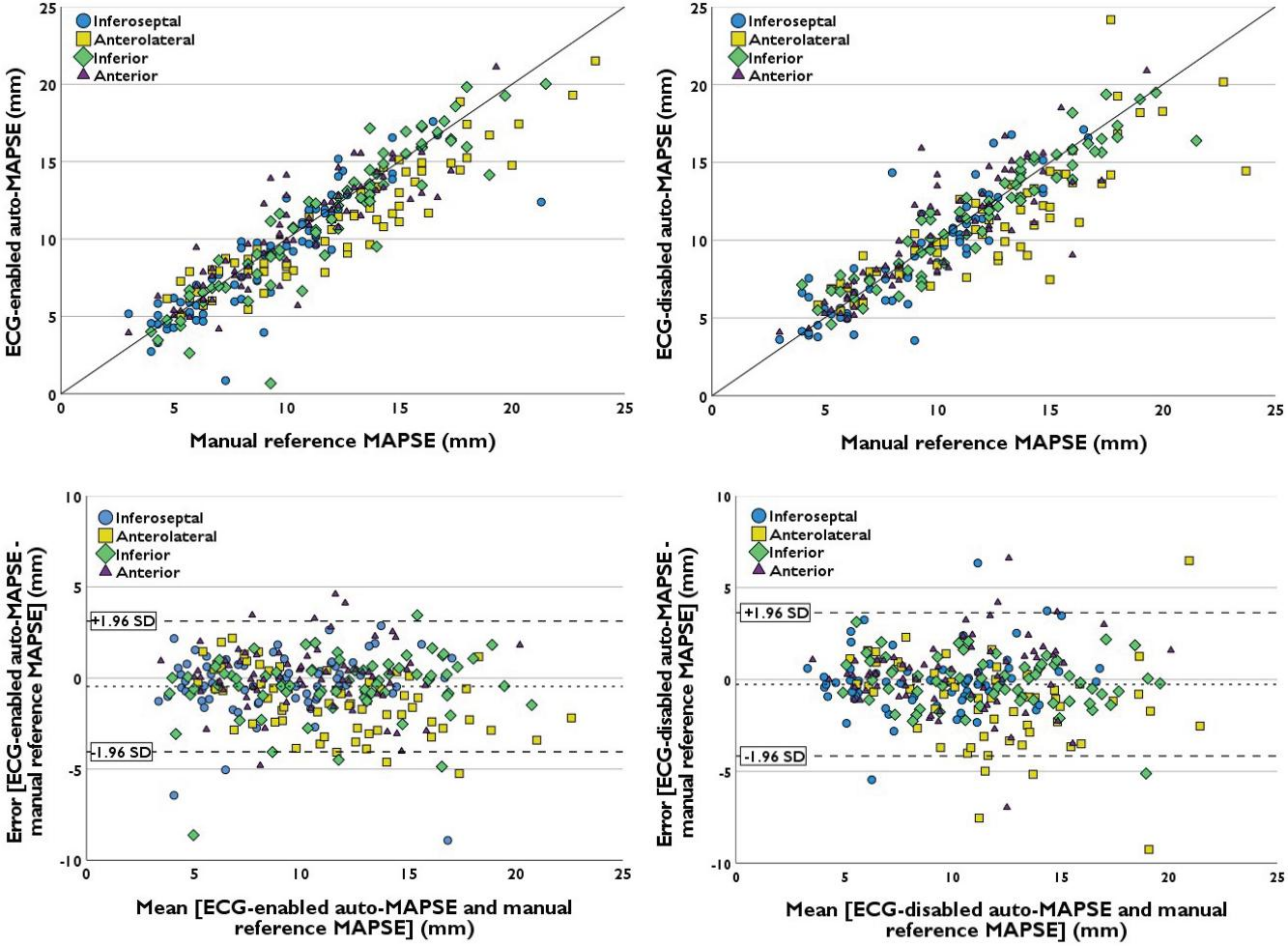
Correlation and Bland–Altman plots of ECG-enabled (left) and ECG-disabled (right) auto-MAPSE compared with manual reference. In the Bland–Altman plots (bottom), the dashed lines represent the bias (middle) and upper and lower 95% LoA (±1.96 SD).

Agreement and inter-rater reliability between auto-MAPSE and manual reference are presented in *Table 4* and *Figure 5*. Compared with manual reference, overall bias [95% LoA] was −0.5 [−4.0, 3.1] mm for ECG-enabled and −0.3 [−4.2, 3.6] mm for ECG-disabled auto-MAPSE. For anterolateral MAPSE, bias was −1.2 and −1.3 mm, respectively, while bias did not exceed ±0.4 mm in the other walls. Overall inter-rater reliability with manual reference did not differ significantly between ECG-enabled and ECG-disabled auto-MAPSE, with an ICC for consistency of 0.90 and 0.88, respectively. Inter-rater reliability between ECG-disabled auto-MAPSE and manual reference was significantly higher in the inferior wall compared with the other individual walls. Inter-rater reliability between ECG-enabled auto-MAPSE and manual reference did not differ significantly between the individual walls.

### Inter-observer

Feasibility, agreement, and inter-rater reliability in the inter-observer subset are presented in *Table 5*.

**Table 5:** Feasibility, agreement, and inter-rater reliability in inter-observer subset (*n* = 30)

Analysis	Observer 2	ECG-enabled auto-MAPSE	ECG-disabled auto-MAPSE	Manual reference
Feasibility, *n* (%)	107 (89%)^a^	101 (84%)	93 (78%)^a^	105 (88%)
Feasibility provided feasible reference, *n* (%)	102 (97%)^b,c^	94 (90%)^b^	88 (84%)^c^	
Bias [95% limits of agreement], mm*	−0.9 [−4.7, 3.0]	−0.6 [−4.3, 3.2]	−0.2 [−4.2, 3.8]	
Intra-class correlation coefficient for consistency (95% confidence interval)*	0.86 (0.80–0.90)	0.88 (0.83–0.92)	0.87 (0.81–0.92)	

Overall results from all 4 walls are displayed. Superscript letters denote pairwise statistically significant differences in feasibility or intra-class correlation between methods. ^a^ *P* = 0.024; ^b^ *P* = 0.049; ^c^ *P* = 0.002 (*P* ≥ 0.05 for all other comparisons).

* Compared with manual reference.

Overall manual inter-observer bias (95% LoA) was −0.9 (−4.7, 3.0) mm with an ICC for consistency of 0.86. Inter-rater reliability between auto-MAPSE and manual reference did not differ significantly from manual inter-observer MAPSE.

## Discussion

We have developed a method for automatic MAPSE estimation by TOE using deep learning. In this clinical validation, we included consecutive TOE examinations performed as part of standard care in a specialized echocardiography laboratory without selection based on image quality. Overall feasibility was >90% for both ECG-enabled auto-MAPSE and manual reference and 82% for ECG-disabled auto-MAPSE. Feasibility in at least two walls was ≥95% for all methods. Inter-rater reliability with manual reference was good with no significant differences between ECG-enabled or ECG-disabled auto-MAPSE or manual inter-observer assessment. Moreover, agreement between auto-MAPSE and manual reference did not differ from manual inter-observer agreement. However, although bias was negligible, the 95% LoA exceeding ±3 mm suggests that auto-MAPSE and manual assessment should not be used interchangeably. Importantly, this also applies to manual inter-observer assessment. Using portable commercial hardware, auto-MAPSE processing time averaged only 0.3 s per cardiac cycle/view, demonstrating that LV systolic function can be monitored in real-time by this method.

Previous work on deep-learning-based auto-MAPSE has been limited to transthoracic echocardiography. A segmentation-based real-time method using only the end-diastole and end-systole frames was imprecise with 95% LoA ±9 mm compared with manual reference.^21^ In healthy volunteers, a method using deep learning for frame-by-frame mitral annular tracking demonstrated better results with 95% LoA ±4 mm compared with manual reference.^22^

CNN performance, i.e. landmark localization, is influenced by CNN architecture and training settings, and by the composition, magnitude, and annotations of the training data set.^23,24^ The magnitude of the training data set represents a reasonable trade-off between annotation workload and amount of training data. We have employed a standard ‘off-the-shelf’ CNN that has been slightly modified for mitral annular localization.^19,20^ Developing a more specific CNN architecture for this task may further improve performance.^24^ In selected cases, the CNN failed to identify the same landmark over time (*Figure 2E*). In addition to increased amount of training data, which is always advantageous in deep learning, this problem may be reduced by deploying more temporal information and/or sequential landmark localization.^22^ Such features may increase feasibility, accuracy, and precision at the cost of processing time.

To discard low-quality data that could potentially return erroneous MAPSE estimates, we applied filter algorithms customized from the pilot validation data set (*Table 1*). Expectedly, this reduced outliers at the cost of feasibility. Optional adjustment of filter settings for the individual patient or clinical setting may be useful. Nevertheless, data quality, and hereby potentially erroneous estimates, is also obvious from the continuous plots of estimated mitral annular location (*Figure 2D* and *E*), which are available to the clinician.

The main cause of non-feasibility was noise at end-diastole following atrial contraction (*Figure 2E*). This phenomenon was likely exaggerated by the foreshortened view of the cardiac chambers, often seen leading to off-centred view of the posterior parts of the left atrium. Specific attention to the challenge and optimization of probe positioning may mitigate this issue.

Mid-oesophageal view deviation from the LV long axis causes overestimation of the true MAPSE by the factor of one over the cosine of the deviation angle. In most cases, this impact is small due to the usually obtained minute angle. Moreover, for monitoring purposes, measurement consistency over time and relative changes in magnitude are more important than absolute values. In this respect, MAPSE estimates are also likely to be less affected by minor probe-to-heart displacement than estimates of ventricular volumes and global longitudinal strain.

Several studies have demonstrated global reduction in MAPSE following myocardial infarction, including walls remote to the infarction.^25–27^ In other words, any regional MAPSE reflects global LV function and not only function in the adjacent wall. In the present data set, mean four- and two-chamber view MAPSEs were strongly correlated, as were anterior and inferior MAPSEs (*Figure 4*). Hence, for monitoring global LV function using MAPSE, one may probably opt for the view with the superior image quality. Moreover, single-wall monitoring from the two-chamber view may also be an option. This may further enhance feasibility (*Table 4*).

We found a larger bias for anterolateral MAPSE compared with other walls. Local differences in systolic excursion around the anterolateral annulus may be quite pronounced, typically increasing with a more lateral annotation. Thus, even minor systematic annotation differences between manual reference and auto-MAPSE may lead to bias. However, at just over 1 mm, this bias has little clinical significance, at least in a monitoring setting, and does not imply that the anterolateral wall should be avoided. Furthermore, although inter-rater reliability between ECG-disabled auto-MAPSE and manual reference was significantly higher in the inferior wall than the other walls, the amount of data behind this is limited, and similar findings were not seen for ECG-enabled analysis. Hence, we advise against generalizing this finding to a common recommendation.

Although CNN training was performed from four- and two-chamber views, auto-MAPSE provides ‘right’ and ‘left’ MAPSE estimates without view distinction. This means that auto-MAPSE may potentially function in non-standardized views, though, importantly, this is yet to be confirmed.

### Limitations

A comprehensive discussion on inherent MAPSE limitations, which also apply to auto-MAPSE, is considered out of scope for this manuscript. MAPSE is insensitive to isolated changes in circumferential strain, which may be caused by alteration of LV circumferential contraction, myocardial compressibility, or geometry. Moreover, the close relationship between MAPSE and global longitudinal strain may be disturbed by mechanical factors such as apical dyskinesia, pericardial adhesions, or external heart compression. However, when such phenomena are either absent or constantly present, in principle, serial MAPSE should be sensitive to changes in LV systolic function.

Occasionally, pronounced lateral and/or elevational translation can make it impossible to align an M-mode or anatomical M-mode to the same mitral annular landmark throughout the cardiac cycle. The same problem does not apply to auto-MAPSE. However, as the auto-MAPSE method estimates total (not strictly longitudinal) mitral annular displacement, lateral translation will lead to an overestimation of MAPSE, which is not corrected by averaging over a full respiratory cycle. If pronounced, this may possibly attenuate auto-MAPSE detection of minor changes in LV function. On the contrary, any axial respiratory movement of the mitral annulus can be corrected by averaging MAPSE estimates over a full respiratory cycle.

By defining end-diastole and end-systole from the peaks of the landmark plots, auto-MAPSE may involve pre- or post-systolic displacement. This may reduce sensitivity to minor alterations in LV systolic function. Image-based detection of end-diastole and end-systole^28^ may alleviate this.

Regardless, frame-to-frame assessment of full cardiac cycles improves precision.^22^

Although recording settings have been mimicked to realistic monitoring situations, the present clinical validation has been performed from single-time-point recordings from an echocardiography laboratory. Auto-MAPSE will return the exact same MAPSE estimate on repeated assessment of the same recording, i.e. perfect reproducibility on identical data. This is a fundamental feature of artificial neural networks provided training mode is disabled and settings are unchanged. However, this study was not designed to determine the precision or least significant change of auto-MAPSE over numerous cardiac cycles and time. Further investigation and validation should be performed in relevant monitoring settings.

## Conclusions

In patients with various heart diseases without selection on image quality, auto-MAPSE had an overall feasibility of 91% with ECG and 82% without ECG. The feasibility of at least two walls was 96% with ECG and 95% without ECG. Inter-rater reliability between auto-MAPSE and manual reference was good, with an overall ICC of 0.90 with ECG and 0.88 without ECG. The agreement between auto-MAPSE and manual reference did not differ from manual inter-observer assessment.

Auto-MAPSE has an ultra-short processing time and may be implemented in modern ultrasound scanners for real-time monitoring of global LV function. The usefulness of real-time application of auto-MAPSE in relevant clinical settings should be investigated, including the potential benefits from improved reproducibility compared with manual assessment.

## Data Availability

The data underlying this study cannot be shared publicly due to limitations in ethical approval and patient consent.

## Lead author biography

Erik Andreas Rye Berg (b. 1981), MD, graduated from Norwegian University of Science and Technology (NTNU) in 2008. He has performed research at the Centre for Innovative Ultrasound Solutions and NTNU since 2016. Since 2017, he has been a consultant in internal medicine at St Olavs Hospital, Orkdal, Norway. He currently holds a position as a fellow in cardiology at St Olavs Hospital, Trondheim, Norway. His main scientific interests are related to myocardial function and valvular heart disease.
